# Structure-guided sequence specificity engineering of the modification-dependent restriction endonuclease LpnPI

**DOI:** 10.1093/nar/gkv548

**Published:** 2015-05-22

**Authors:** Giedrius Sasnauskas, Evelina Zagorskaitė, Kotryna Kauneckaitė, Giedre Tamulaitiene, Virginijus Siksnys

**Affiliations:** Department of Protein–DNA Interactions, Institute of Biotechnology, Vilnius University, Graiciuno 8, LT-02241 Vilnius, Lithuania

## Abstract

The eukaryotic Set and Ring Associated (SRA) domains and structurally similar DNA recognition domains of prokaryotic cytosine modification-dependent restriction endonucleases recognize methylated, hydroxymethylated or glucosylated cytosine in various sequence contexts. Here, we report the apo-structure of the N-terminal SRA-like domain of the cytosine modification-dependent restriction enzyme LpnPI that recognizes modified cytosine in the 5′-C(mC)DG-3′ target sequence (where mC is 5-methylcytosine or 5-hydroxymethylcytosine and D = A/T/G). Structure-guided mutational analysis revealed LpnPI residues involved in base-specific interactions and demonstrated binding site plasticity that allowed limited target sequence degeneracy. Furthermore, modular exchange of the LpnPI specificity loops by structural equivalents of related enzymes AspBHI and SgrTI altered sequence specificity of LpnPI. Taken together, our results pave the way for specificity engineering of the cytosine modification-dependent restriction enzymes.

## INTRODUCTION

5-Methylcytosine (5mC) and its oxidized derivatives, primarily 5-hydroxymethylcytosine (5hmC), are epigenetic marks in eukaryotic cells. The readout of these epigenetic marks is mediated by proteins that specifically recognize modified cytosine variants and strictly discriminate against unmodified cytosine. Structural studies of eukaryotic 5mC/5hmC binding proteins revealed two different strategies for the modified cytosine recognition. Proteins that share the methyl-CpG binding domain as exemplified by MBD (methyl binding domain) proteins and a zinc-finger protein Kaiso, recognize modified C in the context of a Watson–Crick base pair (1–4). The SRA (SET and RING-associated) proteins, exemplified by UHRF1, UHRF2 and SUVH5 methyl-binding domains, extrude the modified base from DNA helix and place it in a protein pocket for discrimination (5–10).

SRA-like domains were recently identified in prokaryotes, where they serve as modules for the modified cytosine recognition by the modification-dependent restriction enzymes that protect host cells from infection by bacteriophages containing methylated, hydroxymethylated or glucosylated DNA. MspJI family enzymes recognize 5mC and 5hmC in various sequence contexts and cut both DNA strands 12/16 nt downstream of the modified cytosine. They are arranged as the N-terminal SRA-like domain and the C-terminal PD-(D/E)XK nuclease domain fusions (11–13). PvuRts1I family enzymes recognize DNA containing 5hmC or glucosylated 5-hydroxymethylcytosine (5ghmC). The optimal substrate for PvuRts1I is a DNA duplex with two 5(g)hmC bases in the opposite strands separated by a ∼20 bp fragment of unmodified DNA. The cleavage occurs at the center of this fragment, e.g. 11/9 nt away from the modified cytosines (14,15). The PvuRts1I and MspJI-family enzymes share PD-(D/E)XK nuclease and SRA-like DNA recognition domains but the domain order is being permuted.

Available data show that the SRA-like fold is a versatile structural module that is used for the recognition of the modified cytosine in a different sequence context (Table 1). The modified cytosine base is extruded outside the DNA helix and accommodated in a pocket of SRA and SRA-like proteins. Subtle structural and size differences of the protein pocket may account for the discrimination of 5mC/5hmC/5ghmC bases by these domains (8,16–17). The recognition of the modified cytosine occurs only in a specific nucleotide context indicating tight coupling between the base flipping and recognition of the surrounding sequence. Structural and molecular mechanisms of sequence recognition by the SRA-like domains are still poorly understood. In the eukaryotic UHRF1-SRA domain specific for the 5mC residue in the CpG sequence context the ‘base flip-promoting’ (‘thumb’) and ‘CpG recognition’ (‘NKR’ finger) loops penetrate into, respectively, major and minor DNA grooves, and make discriminating contacts to the CpG dinucleotide (5–7). In the target sites of the MspJI family enzymes the modified C is embedded into a variable, often degenerate nucleotide sequences that span up to 2 bp upstream and 3 bp downstream of the modified cytosine (Table 1). Readout of the 5′-(mC)NNR-3′ sequence by MspJI REase occurs through the minor groove contacts (18), but the DNA recognition mechanism of other MspJI-like enzymes still has to be resolved. In the present study we report the crystal structure of the N-terminal DNA binding domain of the LpnPI restriction endonuclease (LpnPI-N), which recognizes the modified cytosine in the sequence context 5′-C(mC)DG-3′ (where D – A/T/G, Table 1), and provide mutational/loop-swapping analysis that supports the structural model for the sequence recognition. Our findings pave the way for specificity engineering of the modification-dependent restriction endonucleases.

**Table 1.** Structurally characterized SRA domains and their recognition sequences

Protein	Recognition site^a^	Base modification	PDB ID	References
UHRF1-SRA	5′-(mC)G-3′	5mC, 5hmC	2ZO0, 2ZO1, 3CLZ, 2ZKD, 2ZKE, 2ZKD	(5–7,10)
UHRF2-SRA	5′-(mC)G-3′	5hmC > 5mC	4PW5, 4PW6, 4PW7	(8,10)
SUVH5-SRA	5′-(mC)G-3′	5mC	3Q0B, 3Q0C, 3Q0D	(9)
	5′-(mC)HH-3′			
MspJI	5′-(mC)NNR-3′	5mC, 5hmC	4R28, 4F0Q, 4F0P	(11–12,18)
AspBHI	5′-YS(mC)NS-3′	5mC, 5hmC	4OC8	(11,13)
LpnPI	5′-C(mC)DG-3′	5mC, 5hmC	4RZL	this work, (11)
PvuRts1I	5′-(mC)-3′	5hmC, 5ghmC	4OQ2, 4OKY	(14–15,17,34)
AbaSI	5′-(mC)-3′	5hmC, 5ghmC	4PAR, 4PBA, 4PBB	(15,16)

^a^(mC) – modified cytosine; N – any nucleotide; D – A, T, or G; Y – T or C; S – G or C; R – A or G.

## MATERIALS AND METHODS

### Protein expression, purification and crystallization

The N-terminal LpnPI DNA binding domain LpnPI-N (residues 2–224 of the full-length protein), wt LpnPI and its mutants were expressed and purified as described in (19). In all constructs the first methionine was replaced with a (His)_6_-tag (MGHHHHHHG). According to the mass-spec analysis, the purified LpnPI-N protein did not preserve the N-terminal methionine. The yield of mutant LpnPI variants was comparable to that of the wt enzyme (varied within a factor of two). The folding of all proteins at the secondary structure level was very similar, as demonstrated by the far-UV CD spectra of wt LpnPI and mutants (Supplementary Figure S1).

Protein concentrations were estimated spectrophotometrically using extinction coefficients of 35410/M/cm (LpnPI-N), 49850/M/cm (LpnPI and most mutants / loop-swapping variants), and 51340/M/cm (the loop-swapping variant ‘21AGY’), and are expressed in terms of monomer if not stated otherwise. All extinction coefficients were calculated using the ProtParam tool (http://web.expasy.org/protparam/).

Protein crystallization was performed by sitting drop vapor diffusion method at 291 K. The LpnPI-N protein in 10 mM Tris-HCl (pH 7.5 at 25°C), 200 mM KCl, 0.1 mM EDTA and 0.02% NaN_3_ was concentrated to 5.5 mg/ml (∼220 μM) and mixed with 0.4 volume of the crystallization solution (160 mM (NH_4_)_2_SO_4_, 80 mM HEPES pH 7.5, and 20% w/v PEG 3350). Crystals appeared after 5 days and reached the maximum size in 1 month.

### Data collection and structure determination

Crystal diffraction data for the LpnPI-N protein were collected at 100 K (no extra cryo-protection used) at the MAX II synchrotron I911-3 beamline on a CCD detector. Data were processed with XDS (20), SCALA and TRUNCATE (21). The structure was solved using the molecular replacement protocol of Auto-Rickshaw (22) and the structure of the AspBHI DNA binding domain (PDB ID 4OC8, chain A, residues 6–22, 31–85, 97–208) as the starting model. Molecular replacement procedure during the Auto-Rickshaw run was performed with MOLREP (23), rigid-body refinement was conducted using CNS (24), density modification was performed using PIRATE (21), model was built with ARP/wARP (25), and structure refinement was performed with REFMAC (26) and PHENIX (27). The obtained model was manually rebuilt using COOT (28) and refined using PHENIX (phenix.refine 1.9_1692) (27). During refinement NCS restraints between the two protein subunits present in the asymmetric unit were used. Data collection and refinement statistics are shown in Table 2.

**Table 2.** Data collection and refinement statistics

*Data collection statistics*	
Space group	*P*6_1_
A (Å)	82.037
B (Å)	82.037
C (Å)	152.829
Wavelength	1.0012
X-ray source	MAX II I911-3 beamline
Total reflections	438 186
Unique reflections	33 984
Resolution range (Å)	41.4-2.1
Completeness (%) (last shell)	100 (100)
Multiplicity (last shell)	12.9 (12.8)
I/σ (last shell)	21.5 (5.1)
R(merge) (%) (last shell)	10.1 (52.4)
B(iso) from Wilson (Å^2^)	21.68
*Refinement statistics*	
Resolution range (Å)	41.019–2.10
Reflections work/test	60 566/6585
Protein atoms	3490
Solvent molecules	422
*R*-factor (%)	16.6
*R*-free (%)	19.9
R.M.S.D. bond lengths (Å)	0.010
R.M.S.D. angles (°)	1.092
Ramachandran core region (%)	97.56
Ramachandran allowed region (%)	2.44
Ramachandran disallowed region (%)	0

### Structure analysis

The structures of the N-terminal domain of AspBHI (PDB ID: 4OC8, chain A, residues 2–216), C-terminal domain of PvuRts1I (PDB ID: 4OQ2, chain A, residues 145–290), LpnPI-N (PDB ID: 4RZL, chain A), the protein-DNA complex of UHRF1-SRA (PDB ID: 3FDE, chains ADE), the protein-DNA complex of MspJI (PDB ID: 4R28, chain C, residues 8–263), and apo-MspJI (PDB ID: 4F0Q, chain A, residues 8–263) where overlaid using Multiprot (29). The LpnPI-N interfaces in the crystal were analyzed using the PDBePISA web-server (30), alignments were generated with ESPript (31).

### DNA oligonucleotides

All oligonucleotides were purchased from Metabion. Oligoduplex substrates used in this study are listed in Table 3. Oligonucleotides were 5′-labeled with [γ-^33^P]ATP (Hartmann Analytic) and T4 polynucleotide kinase (Thermo Fisher Scientific). Oligoduplexes were assembled by annealing the corresponding radiolabeled and unlabeled strands.

**Table 3.** Oligoduplex substrates

Oligoduplex	Sequence^a^	Specification^b^
gC(mC)TG	5′-CCGTAGC**5**TGGTCGATCCTAGCTGGTCGCC-3′	Oligoduplex with a standard LpnPI recognition site; the reference substrate in DNA cleavage studies
	3′-GGCATCGGACCAGCTAGGATCGACCAGCGG-5′	
tC(mC)TG	5′-CCGTA***T***C**5**TGGTCGATCCTAGCTGGTCGCC-3′	As gC(mC)TG, but the -2 bp is T:A
	3′-GGCAT***A***GGACCAGCTAGGATCGACCAGCGG-5′	
aC(mC)TG	5′-CCGTA***A***C**5**TGGTCGATCCTAGCTGGTCGCC-3′	The -2 bp is A:T
	3′-GGCAT***T***GGACCAGCTAGGATCGACCAGCGG-5′	
cC(mC)TG	5′-CCGTA***C***C**5**TGGTCGATCCTAGCTGGTCGCC-3′	The -2 bp is C:G
	3′-GGCAT***G***GGACCAGCTAGGATCGACCAGCGG-5′	
gG(mC)TG	5′-CCGTAG***G*5**TGGTCGATCCTAGCTGGTCGCC-3′	The -1 bp is G:C
	3′-GGCATC***C***GACCAGCTAGGATCGACCAGCGG-5′	
gT(mC)TG	5′-CCGTAG***T*5**TGGTCGATCCTAGCTGGTCGCC-3′	The -1 bp is T:A
	3′-GGCATC***A***GACCAGCTAGGATCGACCAGCGG-5′	
gA(mC)TG	5′-CCGTAG***A*5**TGGTCGATCCTAGCTGGTCGCC-3′	The -1 bp is A:T
	3′-GGCATC***T***GACCAGCTAGGATCGACCAGCGG-5′	
gC(mC)AG	5′-CCGTAGC**5*A***GGTCGATCCTAGCTGGTCGCC-3′	The +1 bp is A:T
	3′-GGCATCGG***T***CCAGCTAGGATCGACCAGCGG-5′	
gC(mC)CG	5′-CCGTAGC**5*C***GGTCGATCCTAGCTGGTCGCC-3′	The +1 bp is C:G
	3′-GGCATCGG***G***CCAGCTAGGATCGACCAGCGG-5′	
gC(mC)GG	5′-CCGTAGC**5*G***GGTCGATCCTAGCTGGTCGCC-3′	The +1 bp is G:C
	3′-GGCATCGG***C***CCAGCTAGGATCGACCAGCGG-5′	
gC(mC)TC	5′-CCGTAGC**5**T***C***GTCGATCCTAGCTGGTCGCC-3′	The +2 bp is C:G
	3′-GGCATCGGA***G***CAGCTAGGATCGACCAGCGG-5′	
gC(mC)TA	5′-CCGTAGC**5**T***A***GTCGATCCTAGCTGGTCGCC-3′	The +2 bp is A:T
	3′-GGCATCGGA***T***CAGCTAGGATCGACCAGCGG-5′	
gC(mC)TT	5′-CCGTAGC**5**T***T***GTCGATCCTAGCTGGTCGCC-3′	The +2 bp is T:A
	3′-GGCATCGGA***A***CAGCTAGGATCGACCAGCGG-5′	
gG(mC)TC	5′-CCGTAG***G*5**T***C***GTCGATCCTAGCTGGTCGCC-3′	The -2 bp is G:C and the +2 bp is C:G
	3′-GGCATC***C***GA***G***CAGCTAGGATCGACCAGCGG-5′	
gT(mC)TT	5′-CCGTAG***T*5**T***T***GTCGATCCTAGCTGGTCGCC-3′	The -2 bp is T:A and the +2 bp is T:A
	3′-GGCATC***A***GA***A***CAGCTAGGATCGACCAGCGG-5′	
gA(mC)TA	5′-CCGTAG***A*5**T***A***GTCGATCCTAGCTGGTCGCC-3′	The -2 bp is A:T and the +2 bp is A:T
	3′-GGCATC***T***GA***T***CAGCTAGGATCGACCAGCGG-5′	
gCCTG	5′-CCGTAGC***C***TGGTCGATCCTAGCTGGTCGCC-3′	As gC(mC)TG, but 5mC is replaced with an unmodified cytosine
	3′-GGCATCGGACCAGCTAGGATCGACCAGCGG-5′	

^a^‘**5**’ designates 5-methylcytosine; the DNA regions recognized by LpnPI are underlined; DNA base pairs that deviate from the reference substrate ‘gC(mC)TG’ are shown in ***bold italic*** typeface.

^b^Base pairs upstream of 5mC are numbered −1 and −2; base pairs downstream of 5mC are numbered +1 and +2.

### Mutagenesis

His-tagged full-length LpnPI mutant variants were generated by the QuickChange method (32). The *Escherichia coli* strain ER2566 was used as a transformation host. The mutations were confirmed by DNA sequencing of the entire gene.

### DNA cleavage experiments

DNA hydrolysis reactions were performed by manually mixing radiolabeled oligoduplexes (100–400 nM) with the enzyme (500 nM) in the Reaction Buffer (33 mM Tris-acetate, pH 8.0, 66 mM K-acetate, 10 mM Mg-acetate, 0.1 mg/ml BSA) at 25°C. Samples were collected at timed intervals and quenched by mixing with the loading dye solution (25 mM EDTA, pH 8.0, 95% v/v formamide, 0.01% bromphenol blue). Reaction products were separated by denaturing polyacrylamide gel electrophoresis. The gels contained 20% 29:1 acrylamide/bis-acrylamide with 8 M urea in a standard Tris-borate–EDTA (TBE) buffer, electrophoresis was performed for 1–2 h at 30 V/cm. Radiolabeled DNA was detected and quantified using Cyclone phosphorimager and OptiQuant software. A single exponential was fitted to the substrate depletion data yielding the observed rate constant *k*_obs_. The *k*_obs_ values are reported as an average value from two to four experiments ± 1 standard error of the mean (SEM). The minimal cleavage rate detectable in our experimental setup was 3 × 10^−7^ s^−1^ (corresponds to 2% substrate cleaved during ∼20 h incubation). Wt enzyme cleaved the top (methylated) and the bottom strands of the reference ‘gC(mC)TG’ substrate (Table 3) with comparable rates (data not shown). Cleavage of most other DNA substrates and LpnPI variants was monitored using DNA substrates with the radiolabeled top strand.

## RESULTS

### Overall structure of LpnPI-N

LpnPI recognizes 5mC or 5hmC in the sequence context 5′-C-5(h)mC-(A/T/G)-G-3′, and cuts DNA 10/14 nt downstream from the recognition site (11). It shares protein sequence similarities (Supplementary Figure S2) with structurally characterized AspBHI (42/63% identical/similar aa residues for the N-terminal domains) (13) and MspJI (∼15/33% identical/similar aa residues) enzymes of the MspJI family (12,18).

The structure of LpnPI-N was solved at 2.1 Å resolution (Table 2). The asymmetric unit contains two almost identical protein molecules (RMSD < 0.5 Å over 209 Cα atoms), with slightly larger deviations observed only at the N-termini and two flexible loops (residues 24–26 and 51–54). Both protein subunits make similar contacts in the crystal. The largest contact surface (∼750 Å^2^) is formed between the N- and C-termini of both A and B subunits that encircle the 160–170 β-hairpins of the symmetry related A/B subunits (Supplementary Figure S3). This ‘pinching’ interaction, however, is not functionally important, since LpnPI-N is a monomer in solution (Supplementary Figure S4).

**Figure 1.**
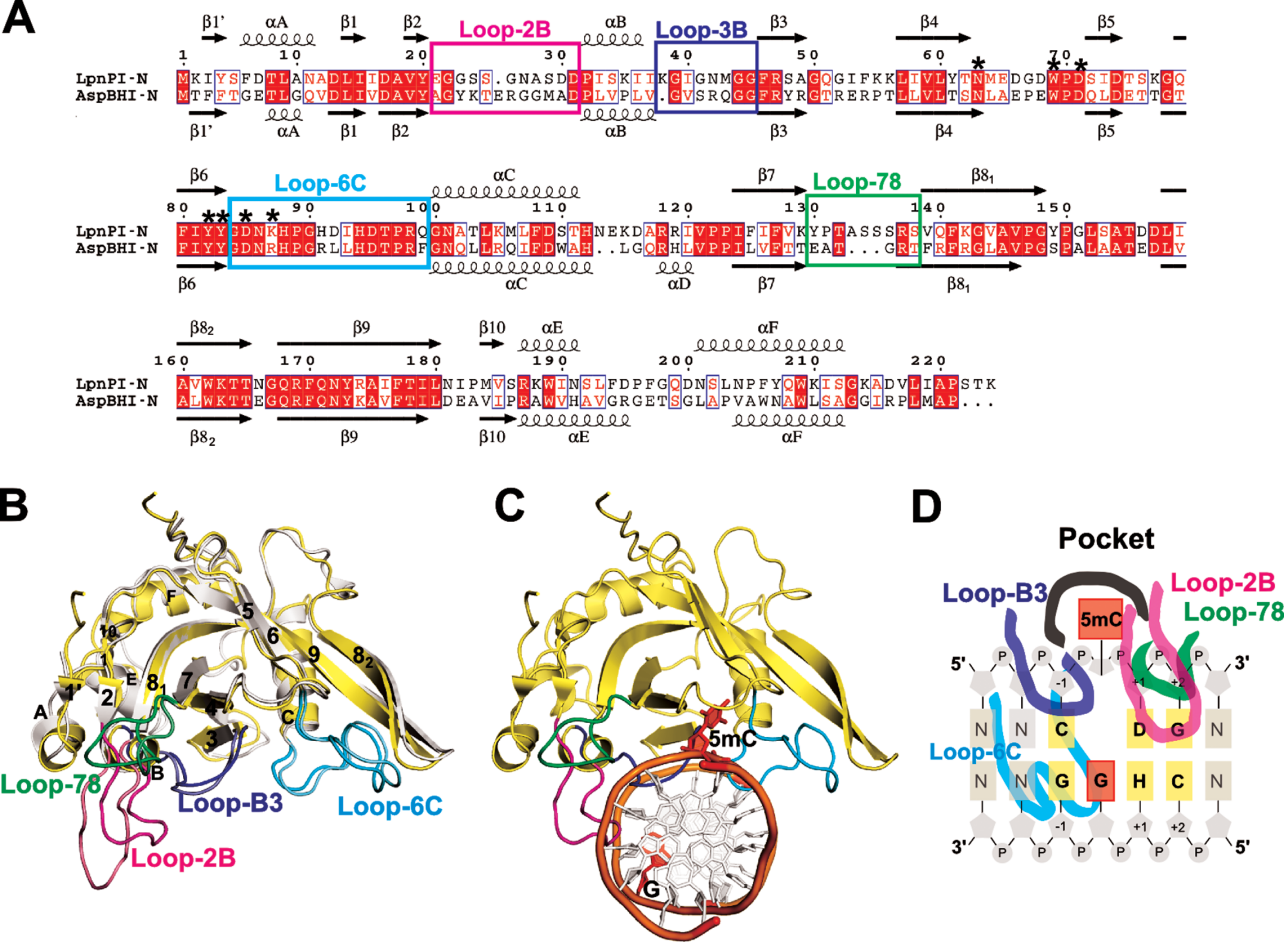
DNA recognition domain of restriction endonuclease LpnPI. (**A**) Sequence alignment of the N-terminal domains of LpnPI (LpnPI-N) and AspBHI (AspBHI-N). Secondary structure elements of LpnPI-N and AspBHI-N are numbered as in (13). Residues that form the flipped-out base binding pocket are marked with asterisks. Alignment was generated with ESPript (31). (**B**) Superimposition of LpnPI-N (in yellow) and AspBHI-N (in white; PDB 4OC8). The putative LpnPI/AspBHI DNA recognition loops are colored as follows: Loop-B3, blue/light blue; Loop-78, green/lime; Loop-2B, magenta/light magenta; Loop-6C, cyan/aquamarine. (**C**) The model of DNA-bound LpnPI-N, based on the crystal structure of DNA-bound UHRF1-SRA (PDB 3FDE). DNA recognition loops are colored as in (B), the flipped cytosine and the orphan intra-helical guanine are shown in red. (**D**) Schematic representation of LpnPI interactions with DNA. Protein loops and the 5mC:G base pair are colored as in panel (C); other bases comprising the LpnPI recognition site are shown in light orange and are numbered from ‘−1’ (the bp upstream of 5mC) to ‘+2’ (the 2nd bp downstream of 5mC).

The overall structure of LpnPI-N is very similar to that of the SRA-like DNA binding domain of AspBHI (RMSD 1 Å over 170 CA atoms, Figure 1A and B). This allowed us to solve the LpnPI-N structure by molecular replacement using the AspBHI-N structure as an initial model (see Materials and Methods for details). The most interesting difference between AspBHI-N and LpnPI-N is the length and conformation of the Loop-2B (residues 21–31, correspond to AspBHI residues 22–33) involved in DNA binding (see below). LpnPI-N is more compact than the corresponding domain of MspJI (224 versus 260 aa). The loops connecting β3–β4, β7–β8 strands and E–F helices are shorter in LpnPI-N by up to 5 aa; nevertheless LpnPI, like AspBHI, contains an 8 aa insertion in the β8 strand that breaks it into two parts (13) (Figure 1A).

### DNA recognition determinants of LpnPI

The DNA-bound structures are available for several eukaryotic SRA domains (5–9) and the MspJI restriction endonuclease (18). Since an overlay of LpnPI-N or AspBHI-N with either the UHRF1-SRA or MspJI co-crystal structures places the DNA molecules and the flipped-out 5-methylcytosine bases in a similar position relative to LpnPI/AspBHI, we will further discuss the models of DNA-bound LpnPI-N and AspBHI-N based on the UHRF1-SRA-DNA structure (Figure 1C and D). We will refer to the DNA base pairs 5′ (upstream) of the flipped cytosine as the ‘−1’ and ‘−2’ positions, and the base pairs 3′ (downstream) of the flipped base as the ‘+1’, ‘+2’ and ‘+3’ positions.

**Figure 2.**
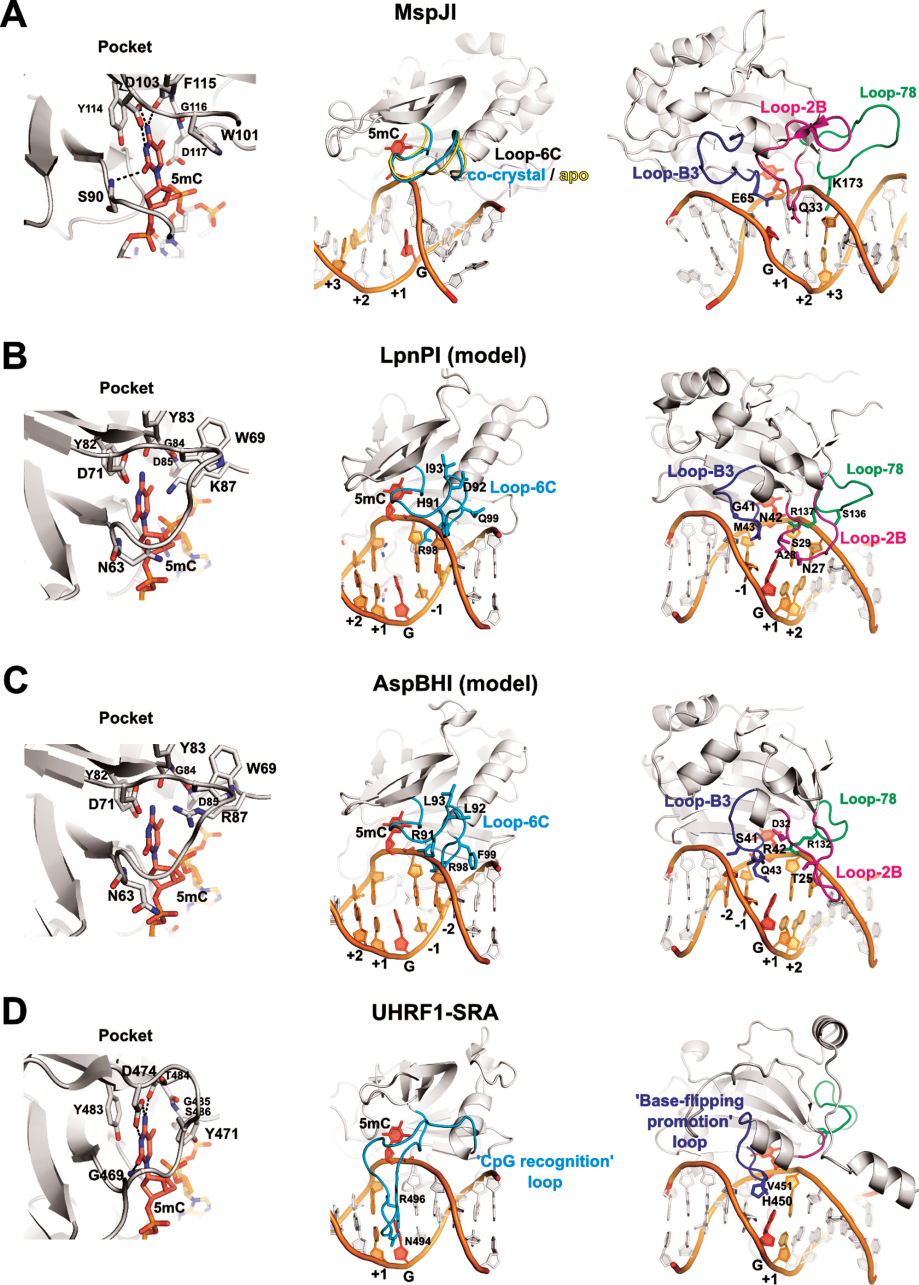
DNA recognition by SRA domains. The structures of the DNA-bound UHRF1-SRA and MspJI, and the apo-structures of MspJI, LpnPI-N and AspBHI-N (PDB ID: 3FDE, 4R28, 4F0Q, 4RZL, 4OC8) were superimposed with MultiProt (29). Equivalent DNA recognition elements in all panels are shown in identical orientation. Left: recognition of the flipped-out base in the protein pocket; center: Loop-6C or ‘CpG recognition’/‘NKR finger’ loop (cyan); right: Loop-B3 or ‘base-flipping-promotion’ loop (blue), Loop-B2 (magenta), and Loop-78 (green). In all panels the flipped-out base and the orphan intra-helical guanine are colored red; other nucleotides comprising the specific recognition sequence of the corresponding protein are colored orange and are numbered from ‘−2’ (the second bp upstream of 5mC) to ‘+3’ (the third bp downstream of 5mC). (**A**) DNA recognition by MspJI. Loop-6C occupies a similar position both in the apo- and the DNA-bound structures and does not make base-specific contacts. Residues Q33, E65, and K173 from the ‘2B’, ‘B3’, and ‘78’ loops, respectively, are close to the DNA bases. (**B** and **C**) The models of DNA-bound LpnPI and AspBHI based on the co-crystal structure of UHRF1-SRA. Loop-C6 and Loop-B3 residues 41–43, 91–93, and 99 are different in LpnPI and AspBHI. LpnPI Loop-2B and Loop-78 residues 27–29 and 136–137 were mutated in the present study; AspBHI Loop-2B residues T25 and D32 are critical for the enzyme function (13); AspBHI Loop-78 residue R132 overlaps with the critical LpnPI residue R137. (**D**) DNA recognition by the SRA domain of the eukaryotic UHRF1 protein. The loops equivalent to Loop-78 and Loop-2B in MspJI-like restriction endonucleases are colored green and magenta, respectively.

The flipped-out cytosine binding pockets are similar in all SRA domains (Figure 2). The cytosine 5-methyl group in MspJI pocket is in van der Waals distance from D117, Y114 and W101 residues, and apparently makes a weak C–H…O hydrogen bond to the carbonyl oxygen of G116. These interactions may serve to distinguish modified cytosine from an unmodified base (18). Equivalent positions in LpnPI and AspBHI are occupied by D85, Y82, W69, and G84 residues. The side walls of the MspJI pocket are formed by the residues W101, Y114 and D117, while D103, S90 and F115 make hydrogen bonds to the Watson-Crick edge of the modified cytosine. Equivalent residues in LpnPI are W69, Y82, D85, K87, D71, N63 and Y83 (W69, Y82, D85, R87, D71, N63, Y83 in AspBHI). Mutation of the AspBHI pocket residues D71, Y82 and D85 to alanine abolished the DNA cleavage activity (13).

An alanine replacement of D71 in LpnPI had a similar effect: the reaction rate decreased more than 1000-fold (Table 4). A more conservative D71N replacement diminished the LpnPI DNA cleavage rate ∼15-fold (Table 4). All DNA cleavage experiments were performed under the optimal reaction conditions (near equimolar enzyme and DNA concentrations; in agreement with the proposed mechanism for the MspJI reaction, which involves simultaneous interaction of the tetrameric enzyme with up to four cognate DNA molecules (12), the LpnPI reactions were much slower under enzyme excess conditions, Supplementary Figure S5). We presume that the observed changes in the DNA cleavage rates under these reaction conditions are due to the altered DNA binding ability of LpnPI.

**Table 4.** Catalytic activity of LpnPI mutants

Mutation	*k*_obs_ (s^−1^)^a^	Activity (%)^b^
wt LpnPI	(3.3 ± 0.8) × 10^−3^	100
5(h)mC binding pocket		
D71A	(1.0 ± 0.3) × 10^−6^	0.03
D71N	(2.0 ± 0. 4) × 10^−4^	6
Loop-2B (contacts downstream of 5(h)mC)		
S25A	(7.0 ± 1.5) × 10^−3^	200
N27A	No cleavage	<0.01
D30A	(1.0 ± 0.3) × 10^−3^	30
Loop-B3 (adjacent to orphan guanine)		
G41S	(1.0 ± 0.1) × 10^−5^	0.3
N42A	No cleavage	<0.01
M43A	(1.0 ± 0.2) × 10^−4^	3
M43Q	(1.6 ± 0.1) × 10^−3^	50
Loop-6C (contacts upstream of 5(h)mC)		
R98A	(1.0 ± 0.6) × 10^−6^	0.03
Loop-78 (contacts downstream of 5(h)mC)		
R137A	(0.7 ± 0.3) × 10^−5^	0.2
S136A	(2.1 ± 0.1) × 10^−3^	60

^a^Oligoduplex DNA cleavage reactions were performed on the ‘gC(mC)TG’ substrate (Table 3) and the observed rate constants *k*_obs_ were determined by single-exponential fits (see Materials and Methods for details). The lowest DNA cleavage rate measured in our assay is 3 × 10^−7^ s^−1^.

^b^The activity is expressed as the ratio *k*_obs_(mutant)/*k*_obs_(wt) × 100%.

In the UHRF1–SRA–DNA complex structure, the vacant space left by the flipped-out base is filled in by the V451 residue from the ‘base flipping-promotion’ or ‘thumb’ loop (5–7) (Figure 2); in MspJI–DNA complex, the E65 residue of the structurally equivalent Loop-B3 (loop between helix αB and strand β3) makes a hydrogen bond to the intra-helical orphaned guanine. The Loop-B3 in LpnPI contains residues N42 and M43 (Figure 2B); M43 (Q43 in AspBHI) is the likely candidate to fill the space left by the flipped-out cytosine, while the N42 (R42 in AspBHI) could make contacts to the orphan guanine or the −1 base pair from the minor groove side. In agreement with this model, the N42A mutation rendered the enzyme inactive, the M43Q mutation had little effect on the enzyme activity, and the M43A mutation decreased the DNA cleavage rate ∼30-fold (Table 4). The glycine residue equivalent to the G41 in LpnPI is conserved in SgrTI and RlaI, but not in AspBHI, which has a serine at this position (Supplementary Figure S2). Interestingly, the G41S replacement reduced LpnPI activity ∼300-fold (Table 4). Presumably, the glycine residue contributes to Loop-B3 flexibility that is important for the LpnPI function.

In the LpnPI–DNA model, the DNA backbone on the 3′ side of the flipped cytosine is contacted by the Loop-78 (residues 130–137 between β7 and β8 strands, Figure 2B). With a little change in a loop conformation, the side chains of S136 and R137 could make base-specific contacts in the major groove 3′ of the extrahelical cytosine; moreover, the K173 residue from an equivalent MspJI loop is the prime candidate for the purine base recognition at the +3 position in the MspJI target sequence (18). DNA cleavage analysis of the alanine replacement mutants S136A and R137A showed that only R137 is critical for LpnPI function (Table 4).

The LpnPI Loop-2B (LpnPI residues 23–31 between β2 strand and αB helix) is positioned in the minor groove 3′ of the flipped cytosine. Structurally equivalent loops are present in both AspBHI and MspJI, but are much shorter in the eukaryotic SRA domains (Figure 2). To probe the role of Loop-2B residues in LpnPI function we made alanine replacements of the polar residues S25, N27 and D30. Only the N27A mutation abolished LpnPI activity, while the other two mutants displayed wt-like activity (Table 4). This is consistent with N27 playing a role in DNA binding/recognition.

The DNA on the 5′ side of the flipped cytosine is approached by the LpnPI Loop-6C (residues 84–99 in LpnPI/AspBHI, and 116–129 in MspJI). An equivalent ‘CpG recognition’ or ‘NKR finger’ loop in the eukaryotic SRA domains is significantly longer (e.g. 484–508 in UHRF1–SRA), adopts a different conformation, and makes base-specific contacts in the DNA major groove (5–7) (Figure 2D). In our LpnPI–DNA model, only the conserved R98 residue of the Loop-6C is within an H-bonding distance to the −1 base. It may also contribute to proper positioning of the adjacent Loop-B3 residues. In agreement with this model, LpnPI mutant R98A was nearly inactive (Table 4).

### The sequence specificity of LpnPI

**Figure 3.**
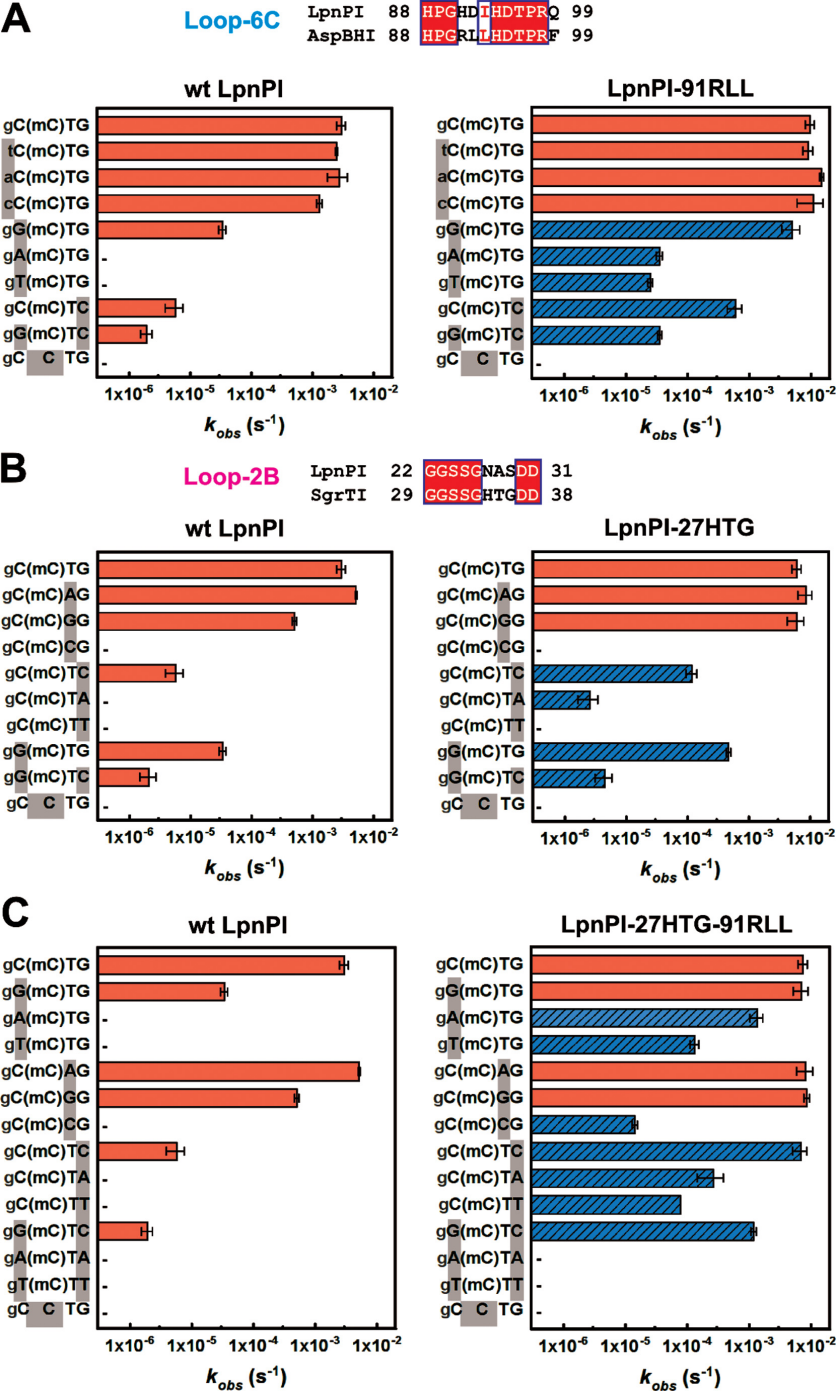
Recognition site preference of LpnPI. Oligoduplex DNA cleavage reactions were performed under standard reaction conditions and the observed rate constants *k*_obs_ were determined by single-exponential fits (see Materials and Methods for details). The recognition sequences of the DNA substrates are shown on the left-hand side of the graphs. ‘(mC)’ stands for 5mC (the last substrate in each graph is the unmethylated control); sequence positions that differ from the reference ‘gC(mC)TG’ substrate are marked with grey boxes; full oligoduplex sequences are listed in Table 3. The reaction rates of LpnPI mutants that show increased cleavage due to loop replacement are marked by blue streaked bars; ‘−’ marks undetectable cleavage (rate lower than 3 × 10^−7^ s^−1^, the starting position of the x-axis). Alignments of the LpnPI/AspBHI Loop-6C and the LpnPI/SgrTI Loop-2B that were replaced in the LpnPI-91RLL and LpnPI-27HTG are shown above panels A and B, respectively. (**A**) Wt enzyme and the LpnPI variant LpnPI-91RLL (Loop-6C replacement) on DNA substrates with variable sequence upstream and downstream of 5mC. (**B**) Wt enzyme and the LpnPI variant LpnPI-27HTG (Loop-2B replacement) on DNA substrates with variable sequence upstream and downstream of 5mC. (**C**) Wt enzyme and the ‘double-swap’ LpnPI variant LpnPI-27HTG-91RLL on DNA substrates with variable sequences upstream and downstream of 5mC.

The LpnPI recognition sequence provided in REBASE (33) is 5′-C(mC)DG-3′ (where mC – modified C and D = A/G/T), though 5′-S(mC)DS-3′ or 5′-(mC)DS-3′ (G>>C) sequence specificities have also been reported (11). To analyze the sequence preference of LpnPI, we measured the LpnPI cleavage rates on a set of oligoduplex substrates (Table 3) that differ from the reference ‘gC(mC)TG’ substrate by 1 or 2 bp. Our results are consistent with LpnPI having a strong preference for the 5′-C(mC)DG-3′ recognition site (cleavage rate constant ∼3 × 10^−3^ s^−1^), albeit ∼90-, ∼500- and 1500-fold slower DNA cleavage was also observed with DNA sites 5′-G(mC)TG-3′, 5′-C(mC)TC-3′, and 5′-G(mC)TC-3′ (rate constants ∼3 × 10^−5^, ∼6 × 10^−6^ and 2 × 10^−6^ s^−1^, respectively, Figure 3A and B). Therefore, the target site for the wt LpnPI can be defined as 5′-(C>>G)(mC)D(G>>C)-3′.

### Sequence specificity engineering of LpnPI

SRA-like DNA binding domains of several MspJI family enzymes have closely related protein sequences (Supplementary Figure S2) and recognize target sites partially overlapping with the 5′-C(mC)DG-3′ target of LpnPI, e.g. AspBHI (5′-YS(mC)NS-3′) and SgrTI (5′-C(mC)DS-3′) (11). Since Loop-6C is the likely candidate for the recognition of the −1 or −1/−2 nucleotides (‘C’ – LpnPI and SgrTI, ‘YS’ – AspBHI) (Figures 1D and 2B, C), we attempted to alter LpnPI sequence specificity by swapping the LpnPI Loop-6C (residues 91–99) with the equivalent loop of AspBHI (Figure 3A). Our expectation was that the resultant LpnPI variant ‘LpnPI-91RLL’ would preferentially cleave DNA substrates with a 5′-YS-3′ dinucleotide in the −1/−2 positions. Indeed, LpnPI-91RLL is more tolerant for a G in the −1 position than the wt enzyme (the ratio of cleavage rates between the 5′-C(mC)DG-3′ and 5′-G(mC)DG-3′ substrates dropped from 90- to 1.5-fold). However, LpnPI-91RLL, like the wt enzyme, has no preference for the −2 base pair (Figure 3A). Loop-6C replacement also increased the tolerance for A and T substitutions in the −1 position: both the 5′-A(mC)DG-3′ and 5′-T(mC)DG-3′ substrates are refractory to the wt enzyme, but are slowly cleaved by LpnPI-91RLL (Figure 3A). Surprisingly, the loop replacement also increased tolerance for substitutions in the downstream part of the recognition site, but to a lesser extent: the rate difference for the 5′-C(mC)DG-3′ and 5′-C(mC)DC-3′ substrates decreased from ∼500- to ∼20-fold (Figure 3A). Thus, the relaxed specificity of LpnPI-91RLL for the −1 position at least in part stems from an overall improvement of enzyme binding to methylated DNA. This may also account for a significant acceleration of the doubly substituted 5′-G(mC)DC-3′ substrate cleavage. Taken these data together, we define the preferred target site for LpnPI-91RLL cleavage as 5′-(S>>W)(mC)D(G>C)-3′.

The sequences of Loop-6C in LpnPI and AspBHI differ by four residues (positions 91–93 and 99, Figure 1A). To identify the residue responsible for the altered LpnPI site preference, we have made LpnPI mutants H91R, D92L, I93L and Q99F, and tested their cleavage activity on the 5′-C(mC)DG-3′ and 5′-G(mC)DG-3′ substrates. We found that the site preference of single point mutants did not change (the rate difference was ∼100-fold for H91R and Q99F mutants, and dropped only to ∼50-fold for the D92L and I93L variants), suggesting that the change in LpnPI-91RLL specificity is due to simultaneous replacement of several residues. In the next step, we also replaced the LpnPI Loop-6C with a corresponding loop from MspJI (residues 123–130), which shows no specificity for the DNA sequence upstream of the modified base. However, the resultant LpnPI variant ‘LpnPI-91VGL’, despite the unperturbed secondary structure, was inactive (Supplementary Figures S1D and S6A).

Our LpnPI-DNA model suggests that Loop-2B contacts DNA to the 3′ side from the modified base (Figures 1D and 2B). To probe the role of Loop-2B residues in DNA recognition, we have made the following LpnPI variants:

- ‘LpnPI-27HTG’, in which three consecutive LpnPI residues (27–29) were replaced with equivalent SgrTI residues (34–36) (Supplementary Figure S2); our expectation was that this would relax the selectivity of LpnPI for the +2 base pair in the 5′-C(mC)DG-3′ sequence and enhance cleavage of the SgrTI recognition sequence 5′-C(mC)DS-3′;
- ‘LpnPI-21AGY’ – the 21–30 LpnPI residues were replaced with 21–31 AspBHI residues (Figure 1A); our expectation was that this would enable cleavage at the AspBHI-like recognition sites with a 5′-NS-3′ dinucleotide at the +1/+2 positions instead of the 5′-C(mC)DG-3′ sequence.

As expected, the LpnPI-27HTG variant had an increased tolerance for a C in the +2 position (the rate difference for the 5′-C(mC)DG-3′ and 5′-C(mC)DC-3′ substrates dropped from ∼500- to ∼50-fold, Figure 3B). The loop replacement also increased the cleavage rate of the 5′-C(mC)TA-3′ substrate, which was refractory to wt LpnPI. Unexpectedly, we have also observed improved cleavage of the 5′-G(mC)DC-3′ substrate with a substitution in the −1 position, as the rate difference for the 5′-C(mC)DG-3′ and 5′-G(mC)DC-3′ substrates decreased from ∼90 to ∼15-fold (Figure 3B). Thus, relaxed recognition of the +2 base pair at least partially may be due to improved non-specific binding to methylated DNA. Taken these data together, we define the preferred target site for LpnPI-27HTG cleavage as 5′-(C>G)(mC)D(G>C>>A)-3′.

We also made point mutations at all three Loop-2B positions that differ between LpnPI and SgrTI (N27H, A28T and S29G). The ratio for the 5′-C(mC)DG-3′ and 5′-C(mC)DC-3′ cleavage rates for the S29G and A28T mutants was ∼200 fold, while the N27H mutant was inactive. Intriguingly, the double mutant N27H+S29G regained full activity, suggesting that the N27H mutation needs extra space or flexibility provided by the S29G mutation. Moreover, the double N27H+S29G mutant displayed DNA cleavage properties akin to LpnPI-27HTG (the ratio for the 5′-C(mC)DG-3′ and 5′-C(mC)DC-3′ cleavage rates dropped to ∼30-fold, data not shown), indicating that N27 and S29 are the key Loop-2B residues involved in DNA binding.

Contrary to LpnPI-27HTG, the LpnPI-21AGY variant preserved wt-like specificity. Unlike AspBHI, it did not tolerate a cytosine at the +1 position, and had a strong preference for a G nucleotide at the +2 position (Supplementary Figure S6B). Involvement of Loop-2B in the recognition of the +1 base therefore seems unlikely. Another plausible candidate for the +1 base pair recognition is Loop-78, which is 3 aa longer in LpnPI than in AspBHI (Figure 1A). To test this hypothesis we have also constructed LpnPI variant ‘LpnPI-133G’, containing a shorter, AspBHI-like Loop-78 version (133–134 LpnPI residues replaced with a glycine, which is equivalent to AspBHI residue G131, Figure 1A). Unfortunately, the resultant LpnPI variant LpnPI-133G was inactive on all substrates tested (data not shown).

Since Loop-6C and Loop-2B act as separate LpnPI DNA binding ‘modules’, we also produced a double-swap LpnPI variant ‘LpnPI-27HTG-91RLL’ containing the AspBHI Loop-6C (relaxes recognition of the -1, and to a lesser extent +2 positions) and the SgrTI Loop-2B (relaxes recognition of the +2, and to a lesser extent −1 positions). Our expectation was that the ‘double-swap’ LpnPI would readily cleave the 5′-SMDS-3′ site. DNA cleavage analysis confirmed this prediction: LpnPI-27HTG-91RLL cleaved 5′-G(mC)DG-3′ and 5′-C(mC)DC-3′ that differ by a single bp from the standard LpnPI recognition site, and the doubly-substituted 5′-G(mC)DC-3′ site, which is poorly tolerated by the wt LpnPI and the ‘single-swap’ variants (Figure 3A-C). Simultaneous substitution of two loops apparently further relaxed LpnPI specificity for the −1 and +2 positions: the ‘double-swap’ LpnPI variant cleaved the 5′-A(mC)DG-3′ and 5′-T(mC)DG-3′ sites only ∼5- and ∼50-fold slower than the standard substrate (the rate differences are >10000- and ∼300-fold for the wt LpnPI and the ‘91RLL’ variant, respectively, Figure 3A); detectable cleavage (∼30–100-fold slower in comparison to the standard 5′-C(mC)DG-3′ substrate) was also observed for the 5′-C(mC)DA-3′ and 5′-C(mC)DT-3′ DNAs, which are both refractory or almost refractory to wt LpnPI and LpnPI-27HTG cleavage (Figure 3B-C). However, no cleavage was detected for DNA substrates 5′-A(mC)DA-3′ and 5′-T(mC)DT-3′, indicating that the presence of two ‘unfavorable’ A:T base pairs in both the -1 and +2 positions is not tolerated (Figure 3C). The recognition sequence of LpnPI-27HTG-91RLL can thus be defined as the combination of 5′-S(mC)(D>>C)-3′ and 5′-(mC)(D>>C)S-3′ recognition sites.

## DISCUSSION

Here, we report the apo-structure of the DNA recognition domain of the cytosine modification-dependent restriction endonuclease LpnPI (LpnPI-N). The overall structure of LpnPI-N is very similar to the DNA binding domain of AspBHI (Figure 1B) (13). Despite the structural similarity, the recognition sequences of LpnPI [5′-C(mC)DG-3′] and AspBHI [5′-YS(mC)NS-3′] differ. This raises a question how different sequence specificity is achieved in the conserved structural scaffold of the MspJI enzyme family. To this end we built a model of DNA-bound LpnPI (Figures 1C and 2B) and performed mutational analysis of LpnPI structural elements involved in DNA contacts, including the modified cytosine binding pocket and four surface loops.

### 5(h)mC binding pocket

The modified cytosine binding pocket is conserved in SRA and SRA-like domains. Typically, the side walls of the pocket are built of aromatic side chains, which make stacking interactions with the extrahelical base, and polar residues, which make cytosine-specific H-bonds to the Watson-Crick edge of the base (Figure 2). Pocket mutations of AspBHI, PvuRts1I and AbaSI proteins severely impaired DNA cleavage activity (13,16–17,34). LpnPI is no exception: the D71A mutation reduced the DNA cleavage rate ∼1000-fold (Table 4). The co-crystal structure of MspJI and the models of DNA-bound AspBHI/LpnPI (13,18) predict that the pocket aspartate (D71 in LpnPI) must be protonated to form a H-bond with the N4 cytosine atom of the flipped-out base (Figure 2B). Since a similar position in another cytosine modification-dependent enzyme PvuRts1I is occupied by an asparagine (N217), we also made the LpnPI mutant D71N. Surprisingly, even this conservative mutation reduced the LpnPI cleavage rate 10-fold (Table 4). Presumably, the structure of the pocket is highly optimized, therefore even slight perturbation of its geometry/H-bonding network has a detrimental effect on enzyme function.

### Loop-B3

LpnPI Loop-B3 approaches DNA from the minor groove side. Structurally equivalent loops in SRA domains provide residues (e.g. V451 in UHRF1-SRA) that fill in the vacant space left by the flipped-out cytosine, and contribute to the recognition of the adjacent base pair (5–7) (Figure 2D). In the MspJI–DNA co-crystal structure a similar position is occupied by E65, which contacts the orphan intra-helical guanine (Figure 2A). Loop-B3 in AspBHI contains residues S41, R42 (both unique to AspBHI, Supplementary Figure S2) and Q43; the same positions in LpnPI are occupied by G41, N42 and M43 (unique to LpnPI). Alanine replacement of the 42th residue in the ‘B3’ loops of both enzymes abolished their activity ((13) and Table 4), suggesting direct involvement of R42/N42 residues in orphan guanine or adjacent base pair recognition. Alanine mutations of residues Q43/M43, which overlap with the UHRF1-SRA V451 residue, were less deleterious, while the LpnPI mutant M43Q displayed wt-like DNA cleavage activity (Table 4). Presumably, the main purpose of the bulky M43/Q43 residues is to fill the space left by the flipped out 5mC rather than make base-specific contacts (Figure 2B).

AspBHI has a preference for the -2 nucleotide to be a pyrimidine (C or T), while LpnPI and other related enzymes accept any nucleotide at this position (11). In our current models of DNA-bound LpnPI and AspBHI (Figure 2B and C) the closest residue to the −2 bp is G41/S41. We speculate that lacking a side chain at the 41th position, LpnPI accepts any nucleotide at the −2 position. In contrast, the same position in AspBHI is occupied by a serine, which could perform pyrimidine/purine discrimination, e.g. *via* a minor groove hydrogen bond to the N3 purine atom in the complementary strand (Figure 2C). In agreement with this model, some AspBHI S41 mutants displayed altered site preference (13). The G41S replacement in LpnPI decreased the cleavage activity ∼300-fold, but did not change the base preference for the −2 position (data not shown). It can not be excluded that other factors, including a subtle difference in the Loop-B3 conformation may contribute to the −2 base pair discrimination by AspBHI.

### Loop-78

Loop-78 approaches DNA downstream of the modified base (Figure 2C). In MspJI, the loop residue K173 is the primary candidate for the recognition of the +3 base pair, where MspJI has a strong preference for a purine (18). Equivalent loops in LpnPI and AspBHI are shorter by five and eight residues, respectively (Supplementary Figure S2). Nevertheless, our current model of DNA-bound LpnPI and mutational data (alanine replacement of Loop-78 residue R137 inactivates LpnPI, Table 4) both suggest that Loop-78 residues contact DNA. Whether these contacts are limited to the DNA backbone, or contribute to the specific recognition of DNA bases (e.g. discrimination of the +1 bp) currently remains unknown, as the replacement of LpnPI Loop-78 with an AspBHI-like shorter loop (LpnPI variant ‘133G’), despite the proper folding of the protein (Supplementary Figure S1C), rendered LpnPI inactive.

### Loop-6C

In the models of DNA-bound LpnPI and AspBHI, Loop-6C approaches the 5′-part of the target sequence from the minor groove side (Figure 2B and C). An equivalent ‘CpG recognition’ or ‘NKR finger’ loop in eukaryotic SRA domains is longer, and makes base-specific contacts in the major groove (Figure 2D) (5–7). Here, we show that replacement of the LpnPI Loop-6C with an equivalent AspBHI loop (four amino acid mutations at positions 91–93 and 99) enables cleavage of the 5′-G(mC)DG-3′ site with a G base in the −1 position, accelerates cleavage of sites with A and T bases in the −1 position, and to a lesser extent improves cleavage of DNA with a C in the +2 position (Figure 3A). This change in site preference could not be emulated by single Loop-6C mutants, indicating that several loop residues contribute to DNA recognition. Intriguingly, three out of four residues replaced in the ‘LpnPI-91RLL’ variant (positions 91–93) in our current model of DNA-bound LpnPI/AspBHI point away from the DNA (Figure 2B and C). Direct contacts to DNA bases by these residues would require a change in Loop-6C conformation similar to that observed in UHRF1-SRA (Figure 2D). However, in MspJI enzyme the Loop-6C occupies the same position both in the apo- and in the DNA-bound structures (12,18) (Figure 2A). The role of the 91–93 and 99 Loop-6C residues in the −1 bp recognition therefore remains undefined: some loop residues may contact DNA bases directly, but this would require an LpnPI/AspBHI-specific change in Loop-6C conformation upon DNA binding; alternatively, Loop-6C residues could contribute to the sequence recognition indirectly through interactions with other protein residues that make direct DNA contacts. An indirect role of Loop-6C residues in DNA recognition may also explain simultaneous relaxation of LpnPI interaction with substrates carrying substitutions both upstream (position -1) and downstream (position +2) of the methylated base. Another important fact is that LpnPI-91RLL, contrary to the donor enzyme AspBHI, had no preference for the −2 bp (Figure 3A). Presumably, recognition of this base pair is performed by another AspBHI structural element, most likely the Loop-B3 (see above).

### Loop-2B

Loop-2B occupies the minor groove on the 3′ side of the modified base (Figure 2A and C). Replacement of the LpnPI Loop-2B with an equivalent loop from SgrTI relaxed the specificity of the LpnPI-27HTG variant for the +2 position, thereby accelerating cleavage of 5′-C(mC)DC-3′ and 5′-C(mC)DA-3′ sites (Figure 3B). Interestingly, cleavage of the 5′-G(mC)DG-3′ DNA, which carries a substitution in the −1 position, was also increased. This suggests that Loop-2B replacement may improve the overall affinity of the enzyme for the methylated DNA. Two out of three LpnPI residues replaced in the ‘27HTG’ variant, namely, N27 and S29, in the current apo-LpnPI/DNA model point away from the DNA and are located closer to the +1 rather to the +2 base pair (Figure 2B). Presumably, upon DNA binding Loop-2B undergoes a conformational change that brings these residues closer to the +2 base pair. The AspBHI Loop-2B is longer by 1 aa and adopts a different conformation (Figures 1B and 2C); its importance for the enzyme function was also confirmed by mutagenesis (T25A and D32A mutations abolished AspBHI activity (13)). Conversely, the glutamine Q33 from the MspJI Loop-2B that contacts the DNA bases 3′ to the flipped cytosine (Figure 2D) is dispensable for MspJI activity (18). This is consistent with MspJI lacking any sequence preference for the +1 and +2 base pairs. An equivalent loop in eukaryotic SRA domains is much shorter and is not involved in base-specific DNA interactions (Figure 2D).

In summary, we show here that LpnPI recognizes the context of the flipped cytosine via several surface loops that act as separate DNA binding/recognition modules. LpnPI is a promising model system for specificity engineering of modification-dependent restriction endonucleases, since it displays a significant plasticity of target site recognition, somewhat reminiscent of homing endonucleases (35). Indeed, though wt LpnPI is most active on the canonical site 5′-C(mC)DG-3′, it also cleaves at alternative 5′-G(mC)DG-3′ and 5′-C(mC)DC-3′, sites albeit at a reduced rate (Figure 3A and B). The LpnPI loop engineering further shifted enzyme preference for alternative recognition sites. Most notably, the ‘double-swap’ LpnPI variant, which carries Loop-2B from AspBHI and Loop-6C from SgrTI, recognizes a shorter target sequence, which can be defined as either 5′-S(mC)(D>>C)-3′ or 5′-(mC)(D>>C)S-3′, and readily cuts the 5′-G(mC)DC-3′ site, which differs from the canonical recognition site 5′-C(mC)DG-3′ by two base pairs (Figure 3C). The relaxed sequence specificity seems to be an intrinsic feature of MspJI family enzymes. From the practical point of view this means that results of a real-life DNA cleavage experiment (% DNA cleaved at particular site) greatly depend on the enzyme/DNA concentrations and the reaction duration. For example, MspJI, AspBHI and LpnPI cleavage sites established under more favorable reaction conditions (with activator oligoduplex) were more ‘relaxed’ than recognition sequences determined under less favorable conditions (no activator duplex) (11). Nevertheless, despite of promiscuous specificity for the target site surrounding the modified cytosine, the SRA-like domain has proved a surprisingly robust module for the modified cytosine DNA recognition: neither the wt LpnPI nor any ‘swap’ or mutant variants showed any activity on unmethylated DNA. The plasticity of the target site recognition intrinsic to the MspJI family enzymes and the stringent discrimination against unmethylated DNA provided by the SRA domain pave the way for engineering of an enzyme specific for the 5mC embedded in any sequence context. Such 5mC-specific enzyme would be a useful tool in genome methylation studies. Significant relaxation of LpnPI specificity presented here is a step towards this goal.

## ACCESSION NUMBER

Coordinates and structure factors of LpnPI-N are deposited under PDB ID 4RZL.

## SUPPLEMENTARY DATA

Supplementary Data are available at NAR Online.

SUPPLEMENTARY DATA
