# Supplementary material for: Structure-guided sequence specificity engineering of the modification-dependent restriction endonuclease LpnPI
Source: Nucleic Acids Res. 2015 May 22;43(12):6144–55. doi: 10.1093/nar/gkv548 (PMC4499157; doi:10.1093/nar/gkv548)
Supplement: SUPPLEMENTARY DATA [file supp_43_12_6144__index.html]

Structure-guided sequence specificity engineering of the modification-dependent restriction endonuclease LpnPI — SUPPLEMENTARY DATA 

# Structure-guided sequence specificity engineering of the modification-dependent restriction endonuclease LpnPI

## SUPPLEMENTARY DATA

- SUPPLEMENTARY DATA
