# Supplementary material for: Structure-guided sequence specificity engineering of the modification-dependent restriction endonuclease LpnPI
Source: Nucleic Acids Res. 2015 May 22;43(12):6144–55. doi: 10.1093/nar/gkv548 (PMC4499157; doi:10.1093/nar/gkv548)
Supplement: SUPPLEMENTARY DATA [file supp_gkv548_nar-00546-h-2015-File005.pdf]

**SUPPLEMENTARY DATA TO:**

# Structure-guided sequence specificity engineering of the modification-dependent restriction endonuclease LpnPI

Giedrius Sasnauskas*, Evelina Zagorskaitė, Kotryna Kauneckaitė, Giedre Tamulaitiene and Virginijus Siksnys*

Department of Protein–DNA Interactions, Institute of Biotechnology, Vilnius University, Graiciuno 8, LT-02241, Vilnius, Lithuania

* To whom correspondence should be addressed. Tel: +370-5-2602111; Fax: +370-5-2602116; Email: gsasnaus@ibt.lt. Correspondence may also be addressed to Virginijus Siksnys. Tel. +370-5-2602108; Fax: +370-5-2602116; Email: siksnys@ibt.lt

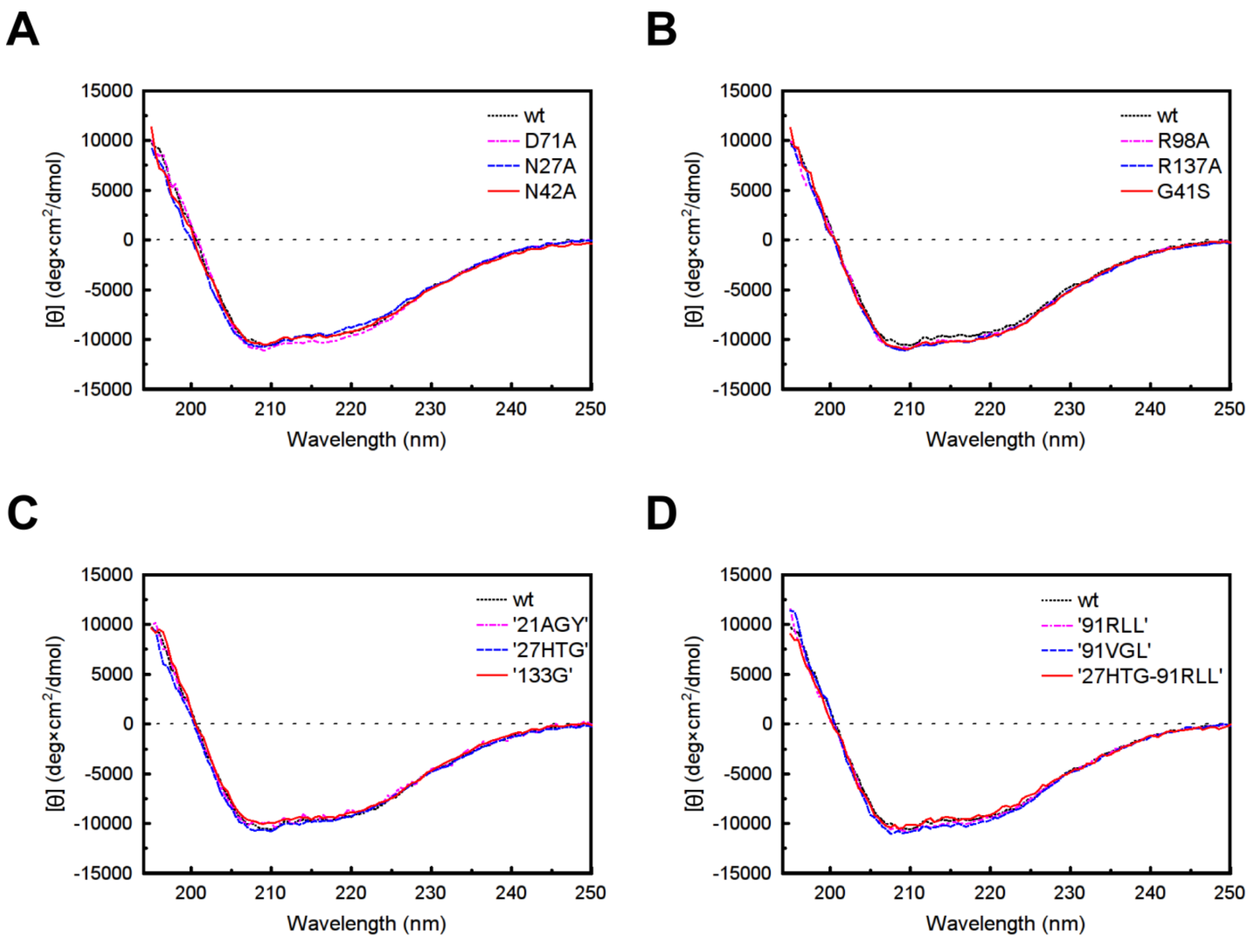


**Supplementary Figure S1. Far-UV CD spectra of wt LpnPI and LpnPI mutants.** Circular dichroism (CD) measurements were carried out on a Jasco J-815 CD spectrometer (Jasco Corporation, Tokyo, Japan). All samples contained 5 μM protein (in terms of monomer, corresponds to 0.23 mg/ml) in 20 mM sodium phosphate (pH 8.0) and 100 mM NaF. The measurements were made at 25 °C using a 1.0 mm pathlength cell. Each 195-250 nm spectrum is the average of three scans run at 50 nm/min, 1 s response time and 1 nm bandwidth. The spectra were corrected for the buffer contribution. The instrument output (ellipticity θ in mdeg) was converted into mean residue ellipticity [θ] (in deg×cm$^2$/dmol) using the formula [θ] = θ×100/(l×c×N), where l – cell pathlength in cm, c – protein concentration in mM, and N – number of protein residues (407 for His-tagged LpnPI).

(A) Wt LpnPI (black dotted line) and LpnPI mutants D71A (magenta dash-dot line), N27A (blue dashed line), N42A (red solid line).

(B) Wt LpnPI (black dotted line) and LpnPI mutants R98A (magenta dash-dot line), R137A (blue dashed line), G41S (red solid line).

(C) Wt LpnPI (black dotted line), LpnPI-21AGY (magenta dash-dot line), LpnPI-27HTG (blue dashed line), and LpnPI-133G (red solid line).

(D) Wt LpnPI (black dotted line), LpnPI-91RLL (magenta dash-dot line), LpnPI-91VGL (blue dashed line), and LpnPI-27HTG-91RLL (red solid line).

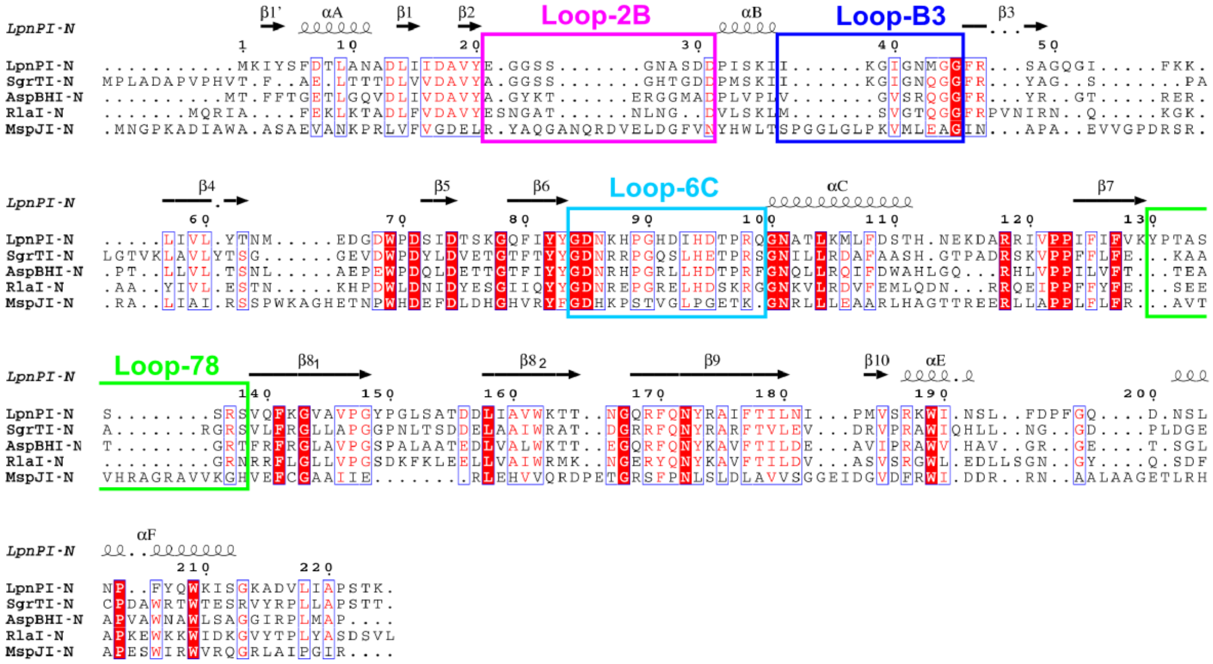


**Supplementary Figure S2. Sequence alignment of the DNA binding domains of LpnPI and related enzymes.** The alignment of the DNA binding domains of LpnPI [recognition site 5′-C(mC)DG-3′, where mC = 5(h)mC], SgrTI [5′-C(mC)DS-3′], AspBHI [5′-YS(mC)NS-3′], RlaI [5′-V(mC)W-3′], and MspJI [5′-(mC)NNR-3′] was generated with T-Coffee Expresso (36) and ESPript (35). The secondary structure elements of LpnPI-N are numbered as in (12). Positions of the LpnPI loops ‘2B’, ‘B3’, ‘6C’ and ‘78’ are shown by magenta, blue, cyan, and green boxes, respectively.

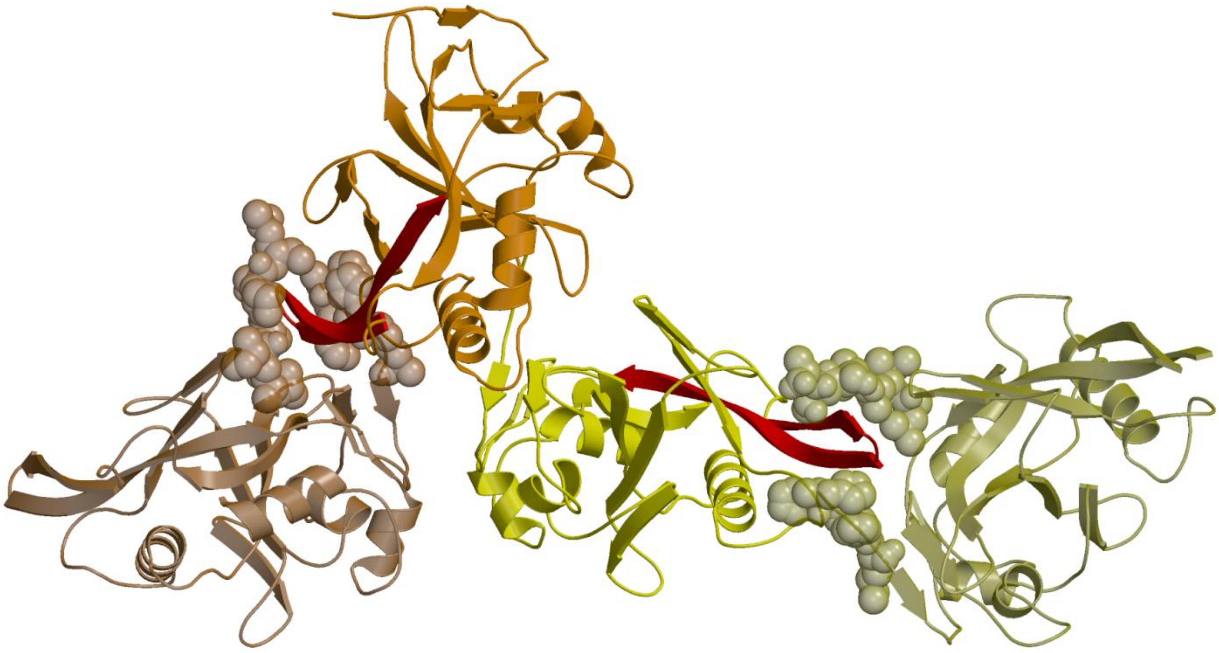

**Supplementary Figure S3. Protein contacts in the crystal.** A (yellow) and B (orange) subunits of the asymmetric unit make similar contacts with the symmetry-related A and B subunits (shown in semi-transparent mode), respectively. β-hairpins, composed of the strands $\beta 8_2$ and β9 (residues 158-180), are coloured red, while the N-termini (residues -2-2) and C-termini (residues 218-224) of the symmetry related subunits are depicted in the spacefill mode.

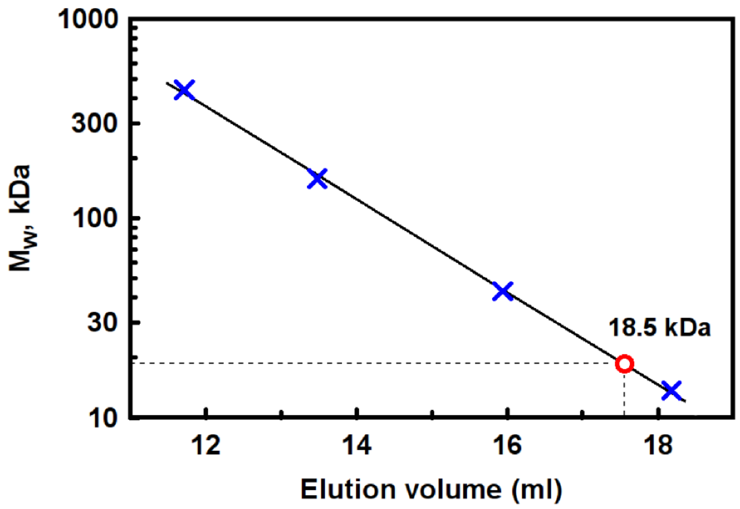


**Supplementary Figure S4. Oligomeric state of LpnPI DNA binding domain.** The apparent MW of LpnPI-N determined by gel-filtration (18.5 kDa, red circle) is close to the theoretic mass of LpnPI-N monomer (25.7 kDa). Gel-filtration was carried out at room temperature on an AKTA Avant system using a Superdex 200 HR column (GE Healthcare) pre-equilibrated with 20 mM Tris-HCl (pH 8.0 at 25°C) and 100 mM KCl. The LpnPI-N sample (4 μM monomer) was prepared in 100 μl of the same buffer. Elution from the column was monitored by measuring absorbance at 280 nm. A calibration line was generated by measuring the elution volumes of a series of standards of known molecular mass (Gel Filtration Calibration Kit from GE Healthcare, blue crosses). The molecular weight of LpnPI-N was calculated by interpolating its elution volume onto the calibration line.

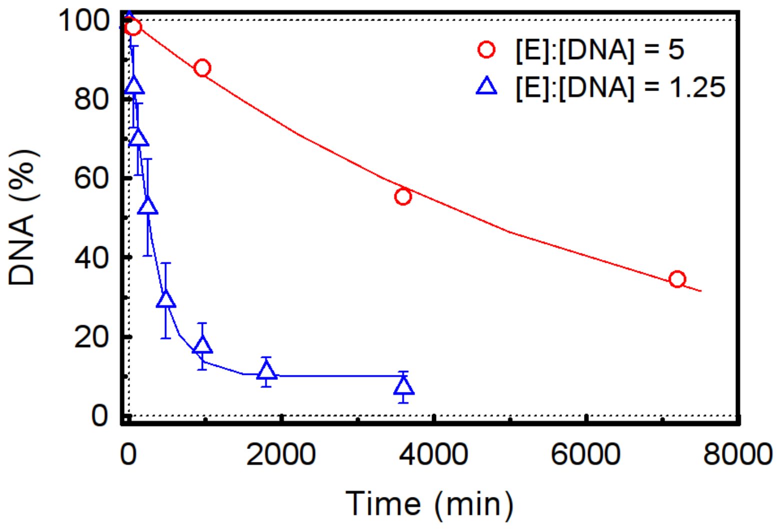


**Supplementary Figure S5. DNA cleavage by wt LpnPI.** DNA cleavage reactions were performed as described in Materials and Methods. The reactions contained either 100 nM (red circles) or 400 nM (blue triangles) nM radiolabeled oligoduplex ‘gC(mC)TG’ (Table 3) and 500 nM LpnPI (monomer). The solid lines represent single-exponential fits to the data. The determined reaction rate constants $k_{obs}$ for the two data sets equal 0.0033 $s^{-1}$ and 0.00015 $s^{-1}$, respectively.

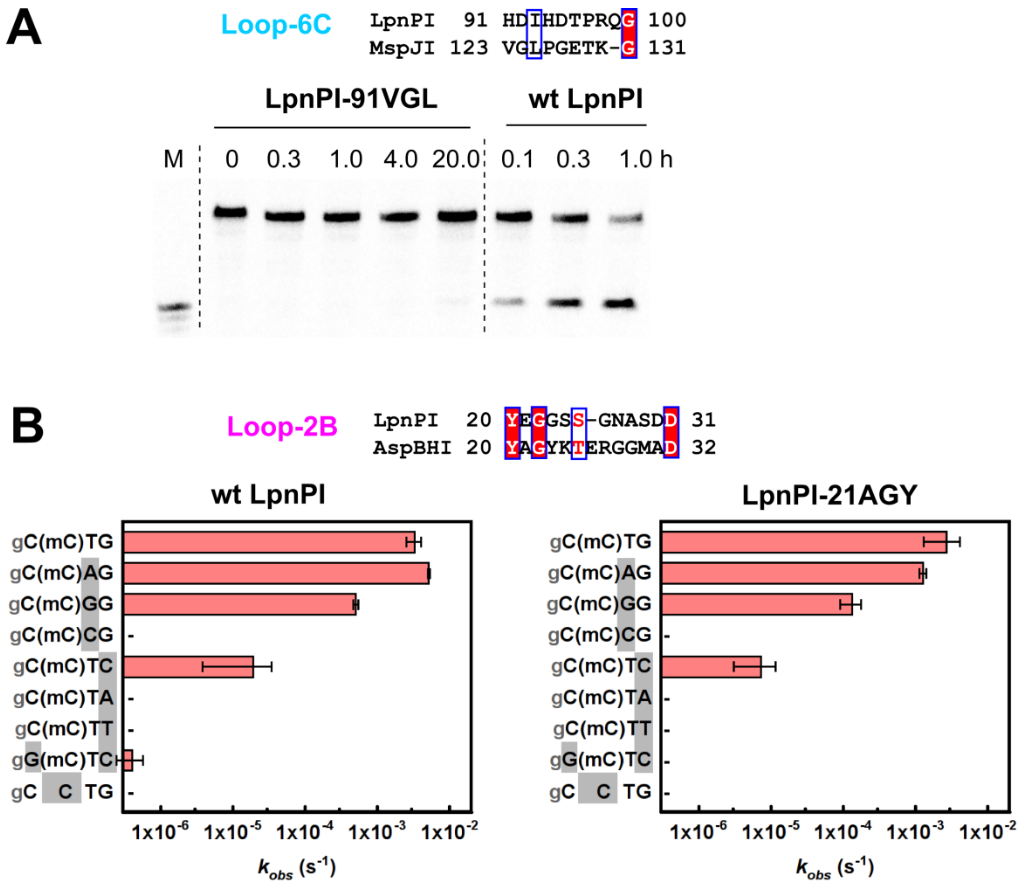


**Supplementary Figure S6. DNA cleavage by LpnPI-91VGL and LpnPI-21AGY.** Oligoduplex DNA cleavage reactions were performed under standard reaction conditions (see Materials and Methods for details). (A) Standard 'gC(mC)TG' substrate (Table 3) cleavage by the LpnPI variant LpnPI-91VGL. Only ~1 % DNA was cleaved after 20 h incubation (LpnPI-91VGL, lane '20'). Gel lane 'M' contains the synthetic DNA cleavage product; gel lanes 'wt LpnPI' contain the same substrate cleaved with wt LpnPI. Alignment of the LpnPI/MspJI Loop-6C residues that were replaced in the LpnPI-91VGL variant is shown above the gel images. (B) Wt enzyme and the LpnPI variant LpnPI-21AGY (Loop-2B replacement) on DNA substrates with variable sequence upstream to 5mC. The recognition sequences of the DNA substrates are shown on the left-hand side of the graphs. '(mC)' stands for 5mC (the last substrate in each graph is the unmethylated control); sequence positions that differ from the reference 'gC(mC)TG' substrate are marked with grey boxes; full oligoduplex sequences are listed in Table 3. '-' marks undetectable cleavage (rate lower than $3\times10^{-7}$ $s^{-1}$, the starting position of the x-axis). Alignment of the LpnPI/AspBHI Loop-2B that was replaced in the LpnPI-21AGY is shown above the graphs.

## Supplementary References

36. Notredame C, Higgins DG, Heringa J (2000) T-Coffee: A novel method for fast and accurate multiple sequence alignment. *J Mol Biol*, **302**, 205–217.
